# 

**Published:** 1919-10-18

**Authors:** 


### THE HOSPITAL

**The Modern Newspaper of Administrative Medicine and Institutional Life.**

Telegraphic Address : OSPEDALE, RAND, LONDON. Telephone Number: 2734 GERRARD

No. 1741, Vol. LXVII. | Saturday, October 18th, 1919. | PRICE TWOPENCE

